# A comparison of sexual desire in opiate-dependent men receiving methadone and buprenorphine maintenance treatment

**DOI:** 10.1186/s12991-019-0249-z

**Published:** 2019-10-22

**Authors:** Anne Yee, Huai Seng Loh, Huai Heng Loh, Shahrzad Riahi, Chong Guan Ng, Ahmad Hatim bin Sulaiman

**Affiliations:** 1 Department of Psychological Medicine, University Malaya Center of Addition Science, Faculty of Medicine, University of Malaya, 50603 Kuala Lumpur, Malaysia; 2 Clinical Academic Unit, Newcastle University Medicine Malaysia, No.1 Jalan Sarjana 1, Kota Ilmu, Educity@Iskandar, 79200 Nusajaya, Johor Malaysia; 3 Department of Medicine, Faculty of Medicine and Health Sciences, University of Malaysia Sarawak, Jalan Dato Muhammad Musa, 94300 Kota Samarahan, Sarawak Malaysia; 4 Department of Pharmacology, Faculty of Medicine, University of Malaya, Kuala Lumpur, Malaysia; 5 Department of Psychological Medicine, Faculty of Medicine, University of Malaya, 50603 Kuala Lumpur, Malaysia

**Keywords:** Sexual desire, Smoking, Methadone, Buprenorphine, Sexual dysfunction

## Abstract

**Background:**

Methadone is an effective therapy for opiate dependence. However, one of the commonest side effects is sexual dysfunction among male patients. Buprenorphine is an alternative to methadone. This study aimed to compare sexual desire among opiate-dependent male patients on buprenorphine (BMT) and methadone maintenance therapy (MMT).

**Methods:**

This cross-sectional study involved 126 male opiate-dependent patient who were tested for total testosterone (TT) and prolactin levels, and were interviewed and completed the Sexual Desire Inventory-2 (SDI-2), Malay language of International Index of Erectile Function (Mal-IIEF-15) and the Malay version of the self-rated Montgomery-Asberg Depression Rating Scale (MADRS-BM) questionnaires.

**Results:**

There were 95 (75.4%) patients on MMT and 31 (24.6%) on BMT. Patients on MMT scored significantly lower in the sexual desire domain (Mal-IIEF-15 scores) (*p* < 0.01), dyadic sexual desire (*p* = 0.04) and TT plasma level (*p* < 0.01) when compared to BMT group after controlling all the confounders.

**Conclusions:**

Patients on MMT are associated with lower sexual desire when compared with patients on BMT. Smoking may further lower testosterone and, hence, sexual desire in those already on methadone.

## Background

Sexual desire (SD) is commonly acknowledged as an individual’s complex motivational state and an interest in sexual objects or activities [1]. It consists of 3 components namely sexual drive, sexual motivation, and sexual wish [2]. Although sexual desire is considered a subjective feeling state which is often influenced by various sociocultural contexts, some experts had broadly defined this aspect of a person’s sexuality as “a psychological state subjectively experienced by the individual as an awareness that he or she wants or wishes to attain a (presumably pleasurable) sexual goal that is currently unattainable” [3], or “a psychobiological energy that precedes and accompanies arousal and tends to produce sexual behavior” [2]. In 2015, a consensus statement from the Fourth International Consultation on Sexual Medicine defined male hypoactive sexual desire disorder, based on DSM-5 [4], as “persistently or recurrently deficient (or absent) sexual or erotic thoughts or fantasies and desire for sexual activity” [5].

Since methadone maintenance treatment (MMT) is a well-recognized effective replacement therapy for opiate dependence [6], for many decades, clinicians have been using methadone with significant success in reducing cases of heroin use [7, 8], criminal activity [9–11], unemployment [10, 12], mortality [13–16] and the transmission of infectious diseases [11, 17, 18]. However, one of the major side effects caused by methadone among male opiate-dependent patients on replacement therapy is sexual dysfunction, such as reduced sexual desire and erectile failure, as it is believed that methadone exerts stronger inhibition effect on sexual desire than heroin [19]. Management for methadone-induced sexual dysfunction remains a challenge for the physicians. Some of the ex opioid-dependent patients relapsed after stopping or reducing their methadone dose. Furthermore, some of them used other illicit drugs to increase their sexual desire [20]. Hence, the physicians need other strategies to manage sexual dysfunction in this group of patients.

Buprenorphine maintenance therapy (BMT), an alternative to MMT, has also been used to treat opioid dependence due to its effectiveness in reducing opioid use. Unlike methadone, buprenorphine is a partial agonist at the µ and κ opioid receptors, and an antagonist at the δ receptors. In a meta-analysis published in 2014 comparing male patients on methadone and buprenorphine, it was shown to have a statistically significant evidence that those males on buprenorphine treatment arm had lower sexual dysfunction compared to the ones on methadone [21].

Previous studies reported that psychiatric disorder, namely depression, is highly prevalent among the MMT population with the prevalence rates range from 19 to 74.3% [22, 23]. Brown et al., [24] and Quaglio et al., [25] reported a statistically significant association between depression and erectile dysfunction among MMT men. However, there were some studies which found no significant associations between depression and erectile dysfunction [6, 26, 27]. Up to date, there are not many studies done to investigate sexual desire among men and even fewer studies to explore the relationship between sexual desire and depression among MMT or BMT patients. Taylor et al. reported that loss of sexual desire was one of the risk factors for relapse and recurrence in major depressive patients who initially responded to cognitive therapy in that 2-year cohort study [28]. Besides, a prospective, 4-week, non-treatment study was conducted on non-depressed men with and without low sexual desire, reported that men with low sexual desire disorder were distressed by their low sexual desire more frequently when compared to men with normal sexual desire [29]. Although previous studies had clearly shown that depression and sexual dysfunction were associated, the relationship between them remained unclear [24–26, 30, 31].

Although low sexual desire is not a life-threatening condition, the prevalence of sexual dysfunction was reported to be in the range of between 21 and 52% [21, 25]. This has potential impacts on the quality of life (QoL) and the intimacy of a relationship [32, 33]. With sexual dysfunction presented in a wide spectrum of conditions, ranging from erectile dysfunction, premature ejaculation to abnormal orgasms, our main aim of this cross-sectional study is to focus on the comparison of sexual desire between opioid-dependent men receiving methadone and buprenorphine maintenance treatment (BMT).

## Methods

### Sample size

According to a previous study [34], a study with 95 MMT and 31 BMT participants would have 80% power to detect the low SD level between them with the effect size of 0.7 at 95% confidence interval [35].

### Study population

This cross-sectional study was conducted in University of Malaya Medical Center and University of Malaya Center of Addiction Sciences, between September 2016 and September 2017. All the opioid-dependent men who received MMT or BMT were approached. Subjects who met all inclusion criteria and none of the exclusion criteria were recruited into the study. Inclusion criteria included: (a) age of more than 18 years with a history of opiate use disorder, (b) have sexual partner, and (c) have been on a stable dosage of either methadone or buprenorphine for more than 8 weeks. Exclusion criteria were: (a) currently on treatment for viral disease such as human immunodeficiency virus (HIV) or hepatitis, (b) currently on treatment for tuberculosis, (c) concurrent use of androgen replacement therapy, phosphodiesterase type 5 inhibitors or any home remedies that could increase sexual desire, (d) concurrent use of psychotropic medications other than methadone or buprenorphine, and (e) any unstable medical condition.

Those who met the inclusion criteria and consented to participate were asked to fill in a semi-structured questionnaire, which included questions on socio-demographic and clinical factors such as, age, ethnicity, education level, employment status, HIV, hepatitis B, hepatitis and other comorbid medical illness. Meanwhile, the Opiate Treatment Index (OTI) was also used to evaluate the drug use, risk-taking behavior, social performance, criminality, health status domain of the MMT or BMT users. In the drug use domain, a Q score is calculated by adding the amount of the most-two-recent drug use and dividing it by the total of the two intervals between used over the past 4 weeks. The higher the scores, the poorer the outcome of that particular domain [36].

All participants were also interviewed face to face by a psychiatrist (First Author) using the Mini International Neuropsychiatric Interview (M.I.N.I) [37], which is a short structured diagnostic interview based on the Diagnostic and Statistical Manual of Mental Disorders, 4th Edition, Text Revision (DSM-IV-TR-IV). In this study, M.I.N.I was used to identify any comorbid psychiatric disorders, namely mood disorders (major depression, bipolar disorder, dysthymia, and suicidality), panic disorder, social anxiety disorder, generalized anxiety disorders, obsessive–compulsive disorder, post-traumatic stress disorder, psychotic disorders, substance dependence and abuse, eating disorders, and antisocial personality in the patients on MMT or BMT.

Sexual desire (SD) was assessed by the Sexual Desire Inventory-2 (SDI-2). This is a 14-item scale measuring the sexual desire in cognitive terms [38]. Four items were concerned about the frequency of desire which scored on an 8-item Likert scale. Ten items were concerned about the intensity of the sexual desire which were reported on a 9-point Likert scale (0 = no sexual desire, 8 = very strong sexual desire). The SDI-2 yields two domain scores: dyadic sexual desire (DSD) and solitary sexual desire (SSD). DSD refers to individuals’ desire for intimacy with other person and SSD refers to individuals’ desire in engaging sexual behavior by oneself. All items are summed up to dictate the total sexual desire (total score = 0 to 109) [38]. This instrument was validated in Malay version (SDI-2-BM) in a sample of 70 benign prostate hyperplasia (BPH) patients and 70 healthy individuals with good internal consistency (DSD, Cronbach alpha = 0.93 and SSD, Cronbach alpha = 0.88) [39].

Erectile Function (EF) was assessed by Malay language of International Index of Erectile Function (Mal-IIEF-15), a 15-item self-report measures five domains of the sexual function which included the erectile function, orgasmic function, sexual desire, intercourse satisfaction, and overall satisfaction over the past 1 month [40, 41]. Each item is rated on a Likert scale ranging from 0 (or 1) to 5, and scores are computed according to each domain. The higher score is corresponding to a better sexual function. Mal-IIEF-15 has good internal consistency of each domain with the Cronbach alpha of 0.74 and higher [41].

The Malay version of the self-rated Montgomery-Asberg Depression Rating Scale (MADRS-BM) was used to assess the severity of the depressive symptoms among MMT and BMT users. This is a self-reported instrument consisting of 9 items which were reported on a 3-point Likert ranging from 0 (no depressive symptoms) to 3 (worst depressive symptoms). Higher scores indicate greater depression. The MADRS-BM exhibited good internal consistency (alpha = 0.78) in the previous study [42].

Lastly, all the participants were tested for total testosterone (TT) and prolactin. All the bloods samples were taken in the morning between 0900 and 1100 h. The blood samples were later used to measure the total TT and prolactin levels using a competitive immunoassay with a direct chemiluminescent technique using ADVIA Centaur (Siemens Healthcare) [43].

### Statistical Analysis

All analyses were conducted with the Statistical Package of Social Sciences, version 22.0 (SPSS, Chicago, IL, USA). Comparisons of the demographic data and clinical characteristics were carried out between the patients on methadone and buprenorphine. Normality was checked using the Shapiro–Wilk test, prior to the analysis of all continuous variables. Independent sample *t* test was chosen for continuous variable with normal distributions, while Mann–Whitney U test was used those which are not. As for all categorical variables, the Chi-square and Fisher exact tests were used. To compare sexual function in patients on MMT and BMT, general linear model (for normally distributed variables) and generalized linear model (for not normally distributed variables) were used while controlling for ethnicity, hepatitis C status, education level, Q scores for tobacco and amphetamines, and social functioning determined by the OTI. Bonferroni multiple testing corrections were used for the pairwise comparisons. Univariate linear regression was performed for the entire study group with DSD as dependent variables and MMT vs. BMT, age, BMI, educational level, HCV infection, OTI scores, and severity of the depression (MADRS-BM score) as independent variables. All the categorical and nominal variables were entered in the regression as dummy variables. *p* < 0.05 was determined as statistical significance using two-sided tests.

## Results

In this study, 150 men who had been receiving MMT and 55 men who had been receiving BMT were approached. A total of 126 male patients who fulfilled the inclusion and exclusion criteria consented to take part in the study. Four MMT patients and two BMT patients refused to participate in the study. The MMT arm consisted of 75.4% (*n* = 95) of the patients (methadone dose = 74.53 mg ± 33.68 mg) while the remaining 24.6% (*n* = 31) made up the BMT group (buprenorphine/naloxone dose = 2.44 mg ± 1.81 mg). Demographic and clinical details of the study subjects are shown in Table 1. The significant differences between patients in both MMT and BMT groups were education level (*p* < 0.01); hepatitis C status (*p* < 0.01); OTI Q scores for tobacco (*p* < 0.01), social functioning and health (*p* < 0.01), and total MADRS-BM score (*p* < 0.01) (Table 1).

**Table 1:** Demographic and treatment characteristics of all male participants

	MMT (*n* = 95)	BMT (*n* = 31)	*df*	*χ*^2^, *Z*, *t*	*p* value
Age, years, mean ± SD	43.51 ± 9.82	41.60 ± 9.80	123	0.93	0.37
Daily dose, mean ± SD	74.53 ± 33.68	2.44 ± 1.81	–		
Duration of MMT or BMT, mean ± SD	50.07 ± 37.86	64.47 ± 44.21	123	− 1.74	0.08
BMI, mean ± SD	23.64 ± 4.80	23.46 ± 4.23	123	0.19	0.84
Ethnic group, *n* (%)					
Malay	87 (91.6)	28 (90.3)	3	4.29	0.22
Chinese	7 (7.4)	1 (3.2)			
Indian	1 (1.1)	1 (3.2)			
Others	0	1 (3.2)			
Religion, *n* (%)					
Islam	87 (91.6)	29 (93.5)	3	1.43	0.69
Christian	1 (1.3)	1 (3.2)			
Buddha	6 (6.3)	1 (3.2)			
Hindu	2 (2.7)	0			
Others	1 (1.1)	0			
Education level, *n* (%)					
No education	1 (1.1)	0	3	16.55	< 0.01**
Primary	9 (9.5)	12 (38.7)			
Secondary	75 (78.9)	19 (61.3)			
Tertiary	10 (10.5)	0			
Employment, *n* (%)	84 (88.4)	30 (96.8)	1	1.89	0.29
Family history of drug use, *n* (%)	21 (23.1)	2 (6.5)	1	4.18	0.06
HBV, *n* (%)	4 (4.2)	0	1	1.35	0.57
HCV, *n* (%)	43 (45.3)	2 (6.5)	1	15.34	< 0.01**
OTI, mean ± SD					
Q scores of drugs use^a^					
Tobacco	12.69 ± 9.48	15.75 ± 6.21	123	− 3.23	0.001**
Alcohol	0.01 ± 0.21	0	123	− 0.79	0.43
Benzodiazepine	0.02 ± 0.10	0.0007 ± 0.004	123	− 1.14	0.89
Marijuana	0.0005 ± 0.004	0	123	− 0.85	0.39
Amphetamines	0.002 ± 0.01	0	123	− 1.41	0.16
Heroin	0.014 ± 0.104	0	123	− 1.41	0.16
HIV risk-taking	5.16 ± 2.81	5.23 ± 2.61	123	− 0.34	0.73
Criminality	0	0	–		
Social functioning^a^	5.71 ± 5.34	1.65 ± 2.56	123	− 4.43	0.001**
Health^a^	0.46 ± 0.80	0.10 ± 0.31	123	− 2.65	0.008**
M.I.N.I. psychiatric disorder(s), *n* (%)	15 (15.8)	4 (12.9)	5	8.05	0.153
Current MDD	1 (0.8)	0	1	0.33	1
Past history of MDD	2 (1.6)	0	1	0.66	1
Dysthymia	1 (0.8)	0	1	0.33	1
Panic disorder	1 (0.8)	0	1	0.33	1
GAD	0	2 (1.6)	1	6.23	0.06
ASD	12 (12.6)	2 (11.1)	1	0.90	0.515
Total MADRS-BM score, mean ± SD	3.11 ± 4.00	0.57 ± 1.63	103.07	− 4.22	< 0.01**

MMT, methadone maintenance treatment; BMT, buprenorphine maintenance treatment; BMI, body mass index; HBV, hepatitis B; HCV, hepatitis C; OTI, Opioid Treatment Index; M.I.N.I., Mini International Neuropsychiatric Interview; MDD, major depressive disorder; GAD, Generalized anxiety disorder; ASD, antisocial disorder; MADRS-BM, Malay version of self-rated Montgomery-Asberg Depression Rating Scale; df, degrees of freedom; SD, standard deviation; *t*, *t*-test; *χ*^2^, Chi square test; *Z*, *z*-test

* *p* < 0.05, **p < 0.01

^a^Based on Mann–Whitney test

Comparison of differences was done by using multivariate analysis of covariance in the SDI-2-BM, Mal-IIEF-15 scores, total testosterone (TT) and prolactin between the MMT and BMT groups. This measure also took into account the control of confounders such as education level, hepatitis C status, total MADRS-BM score, Q scores for tobacco, social functioning health OTI domains with pairwise comparisons using Bonferroni multiple testing corrections. Our study revealed that patients on MMT scored statistically significantly lower in the sexual desire domain (Mal-IIEF-15 scores) (*p* < 0.01), DSD (*p* = 0.04) and TT plasma level (*p* < 0.01) when compared to the BMT group after controlling all the confounders (Table 2).

**Table 2:** Comparison of mean Mal-IIEF-15 domain scores, SDI-2-BM, total testosterone and prolactin in patients with sexual partners in the MMT and BMT groups

	MMT (*n* = 95)	BMT (*n* = 31)	Mean difference^a^	*p* value^b^
	mean ± SD	mean ± SD		
Mal-IIEF-15 domain				
Erectile function	21.09 ± 7.91	23.33 ± 7.71	− 2.23	0.23
Orgasmic function^c^	7.21 ± 4.97	7.15 ± 2.93	0.06	0.96
Sexual desire	6.14 ± 1.76	7.59 ± 1.54	− 1.46	0.001**
Intercourse satisfaction	8.59 ± 3.67	9.60 ± 3.54	− 1.01	0.25
Overall satisfaction	7.09 ± 2.31	7.93 ± 2.14	− 0.11	0.84
Total	48.87 ± 17.10	59.60 ± 15.97	− 4.75	0.23
DSD	27.74 ± 10.95	33.39 ± 11.18	− 5.66	0.04*
SSD	5.25 ± 5.00	4.15 ± 3.74	1.09	0.37
Total testosterone (nmol/L)	12.46 ± 7.64	18.45 ± 9.40	− 5.99	0.005**
Total testosterone (ng/dL)	359.37 ± 220.35	532.13 ± 271.11		
Prolactin (μIU/mL)	162.12 ± 102.69	165.20 ± 67.71	− 2.77	0.91

BMT, buprenorphine maintenance treatment, MMT, methadone maintenance treatment; Mal-IIEF-15, Malay version of the International Index of Erectile Function 15; SDI-2-BM, Malay version of the Sexual Desire Inventory-2; DSD, dyadic sexual desire; SSD, solitary sexual desire; SD, standard deviation, nmol/L, nanomoles per liter; ng/dL, nanograms per decilitre; μIU/mL, macro international units per milliliter

* *p* < 0.05, ** *p* < 0.01

^a^Adjusted mean difference with education Level, hepatitis C status, total Malay version of self-rated Montgomery-Asberg Depression Rating Scale (MADRS-BM) score, social functioning total score, health total score and Q score for tobacco as covariates

^b^Adjusted for multiple comparisons using Bonferroni correction

^c^A generalized linear model approach was used because the variables were not normally distributed

### Associated factors and the sexual desire

Only MMT vs BMT groups (*β* = 0.31, Adj *R*^2^ = 0.05, *p* < 0.001) and OTI Q score for tobacco (*β* = − 0.26, Adj *R*^2^ = 0.10, *p* = 0.01) were associated with DSD in linear regression by using stepwise method. The MMT vs BMT groups were entered as a dummy variable model with MMT = 0 and BMT = 1. Age, education level, hepatitis C status, OTI Q scores for alcohol, heroin, stimulant, cannabis use, social functioning and health domain, and severity of the depression showed no significant associations with DSD.

## Discussion

Findings from our study show lower mean score for sexual desire (*p* = 0.001) among male opiate-dependent patients treated with MMT compared to those on BMT. This indicates that patients on BMT achieved a higher sexual desire score after controlling for all possible confounders.

In our study, the total testosterone level for male patients in the MMT group appeared significantly lower (12.46 ± 7.64) when compared to that of the BMT group (18.45 ± 9.40) (*p* = 0.005). This could be explained by Smith and Elliot’s study in 2012, where they found human and animal data indicating that opioid acted at different sites in the hypothalamus–pituitary axis, leading to an endocrine dysfunction known as opioid associated androgen deficiency (OPIAD) [44]. All pituitary hormones were decreased, such as luteinizing hormone, follicle stimulating hormone, oxytocin, estradiol, and obviously the reduction of testosterone hormone resulted in hypogonadism. In the same year 2012, Heidari et al. also suggested that lower risk of OPIAD was observed in those who were treated with buprenorphine, compared to methadone due to the offset of hypothalamic–pituitary axis inhibition related to κ-opiate receptor antagonist activity [45], though it was a study conducted on animals. Buprenorphine had minimal influence on testosterone levels [27], as the antagonism of buprenorphine κ-opiate receptor might have possibly counteracted on the μ-opiate receptor-mediated depression of the gonadal axis. In contrast, the pharmacodynamic effects of methadone on sexual behavior are similar to those anti-androgenic effects [46] presenting with symptoms of testosterone deficiency such as fatigue, weakness, mood disturbances, and decrease in libido and sexual function [47].

Chronic exposure to tobacco by long-term smoking can lead to decrease in serum testosterone level, subsequently causing erectile dysfunction in males [48]. In our study, those patients in the MMT group scored statistically significantly lower in the sexual desire domain (Mal-IIEF-15 scores) (*p* < 0.01), DSD (*p* = 0.04) and TT plasma level (*p* < 0.01) when compared to BMT group after controlling all the confounders. This means that if smoking habit continues, there is a higher chance of these male opiate-dependent patients developing even lower testosterone levels, consequently leading to sexual dysfunction. Clinicians should, therefore, play an active role in advising and helping all male patients on methadone therapy to cease smoking, to reduce sexual dysfunction among them.

In a previous study, it was found that sexual desire does not solely depend on its biological component, and its psychological component is influenced by the interpersonal state (presence or absence of sexual partner) and social context [49]. However, in our study, the severity of depression and social functioning were not statistically significant post-linear regression. This implies that the low sexual desire was exclusively caused by methadone in this group of patients.

Our study is not without its limitations. First, this is a cross-sectional study and recall bias might have taken place as we had to rely on self-report data based on patient’s memory. Second, in a relatively conservative society in Malaysia, participants might have concealed their true feelings because they felt uncomfortable to reveal everything to the researchers, hence leading to the possibility of response bias. Third, all male opioid-dependent patients were recruited from a university hospital methadone clinic and the findings may not be generalized to the population with substance misuse. Fourth, the clinical severity of opioid dependence, such as frequency and duration of opioid use, prior to the treatment was not considered in this study. However, it is worth noting that our study carries strengths such as the inclusion of a sample size determined by power calculation, exclusion of mental and physical illness associated with sexual dysfunction, and the use of validated instrument to measure sexual dysfunction.

## Conclusions

The present data suggest that the use of methadone is associated with lower sexual desire when compared with the use of buprenorphine in opioid-dependent patients, but larger studies are needed to confirm the present findings. These results highlight the importance of awareness among clinicians who treat sexual desire disorder when making treatment decisions in this population. Reducing this ubiquitous complication arising from replacement therapy will, in the long run, greatly influence the positive prognosis of their treatment compliance and drug dependence.

## Data Availability

All relevant data are within the paper. The datasets used and/or analyzed during the current study are available from the university of the corresponding author on reasonable request.

## Abbreviations

BMT: buprenorphine maintenance therapy
MMT: methadone maintenance therapy
TT: total testosterone
SDI-2: Sexual Desire Inventory-2
Mal-IIEF-15: Malay language of International Index of Erectile Function
MADRS-BM: Malay version of the self-rated Montgomery-Asberg Depression Rating Scale
DSD: dyadic sexual desire
SD: sexual desire
QoL: quality of life
OTI: Opiate Treatment Index
SSD: solitary sexual desire
OPIAD: opioid-associated androgen deficiency
